# Ninjurin 1 contributes to TLR-induced inflammation in endothelial cells

**DOI:** 10.1186/cc11731

**Published:** 2012-11-14

**Authors:** C Jennewein, K Zacharowski

**Affiliations:** 1 Hospital of the Goethe-University Frankfurt, Clinic of Anaesthesiology, Intensive Care and Pain Therapy, Frankfurt, Germany

## Background

Nerve injury induced protein 1 (Ninjurin 1 (Ninj1)) was first identified in Schwann cells and neurons contributing to cell adhesion and nerve regeneration. Recently, the role of Ninj1 has been linked to inflammatory processes in the central nervous system where functional repression reduced leukocyte infiltration and clinical disease activity during experimental autoimmune encephalomyelitis in mice [1]. But Ninj1 is also expressed outside the nervous system in various organs such as the liver and kidney as well as on leukocytes [2,3]. Therefore, we hypothesized that Ninj1 contributes to inflammation in general; that is, also outside the nervous system, with special interest in the pathogenesis of sepsis.

## Methods

Ninj1 was repressed by transfecting HMEC-1 cells, a human dermal microvascular endothelial cell line with siRNA targeting Ninj1 (siNinj1) or a negative control (siC). Subsequently, cells were stimulated with 100 ng/ml LPS (TLR4 agonist), 3 μg/ml LTA (TLR2 agonist) or 100 n/ml poly(I:C) (TLR3 agonist) for 3 hours. The inflammatory response was analyzed by real-time PCR. In addition, transmigration of neutrophils across a HMEC-1 monolayer was measured using transwell plates (pore size 3 μm).

## Results

**Figure 1:**
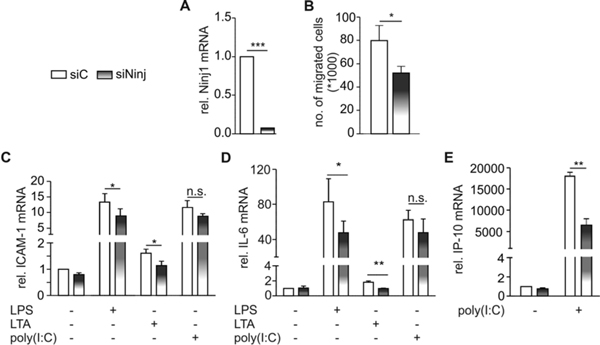
**Effect of Ninj1 on TLR-induced inflammation**.

Repression of Ninj1 by siRNA reduced Ninj1 mRNA expression in HMEC about 90% (Figure 1A). Reduced Ninj1 expression decreased neutrophil migration to 62.5% (Figure 1B) and TLR signaling. In detail, knockdown of Ninj1 significantly reduced TLR-2 and TLR-4 triggered expression of ICAM-1 and IL-6 (Figure 1C,D) while poly(I:C)-induced expression was only slightly reduced. To analyze a more specific TLR-3 target, we measured IP-10 mRNA expression, which was also significantly reduced in siNinj1-transfected cells (Figure 1E).

## Conclusion

Our *in vitro *data strongly indicated that Ninj1 is involved in regulation of TLR signaling and therewith contributes to inflammation. *In vivo *experiments will clarify its impact on systemic inflammation.
